# Biomarkers for Cancer Immunotherapy: Advances, Challenges, and Clinical Applications

**DOI:** 10.3390/ijms27219882

**Published:** 2026-11-04

**Authors:** Adeel Ahmed, Faridul Haq

**Affiliations:** 1 Institute of Brain Science and Brain-Inspired Research, Shandong First Medical University & Shandong Academy of Medical Sciences, Jinan 250117, China; abbasiad2022@gmail.com; 2 Department of Neurosurgery, Eulji University School of Medicine, Daejeon 3428, Republic of Korea

**Keywords:** cancer immunotherapy, biomarkers, PD-L1, tumor mutational burden, microsatellite instability, circulating tumor DNA, tumor microenvironment, immune profiling, precision oncology, resistance mechanisms

## Abstract

Cancer immunotherapy has revolutionized oncology by harnessing the immune system to recognize and eliminate malignant cells. Despite remarkable clinical successes, a substantial proportion of patients fail to respond or develop resistance, highlighting the urgent need for reliable biomarkers to guide patient selection, predict therapeutic outcomes, and monitor treatment responses. This review addresses both the emerging and established biomarkers for cancer immunotherapy. These include molecular, cellular, and systemic biomarkers within tumor tissues, blood, and the tumor microenvironment. The principal ones include immune checkpoint-related biomarkers (e.g., programmed death-ligand 1 (PD-L1) expression), tumor mutational burden, microsatellite instability, neoantigen profiling, circulating tumor DNA, and immune cell signatures. We grade each biomarker by analytical validity, clinical validity, clinical utility and regulatory readiness; place it in one of four evidence tiers; and outline the steps needed to bring candidate markers into the clinic. Recent developments in multi-omics technologies, artificial intelligence, and single-cell analysis are accelerating biomarker discovery, providing a clearer understanding of tumor–immune interactions. Furthermore, the integration of dynamic biomarkers is critical for optimally refining therapeutic strategies for real-time monitoring and detection of resistance mechanisms. This review aims to provide a worldwide overview of present achievements, challenges, and future opportunities in biomarker research and ultimately enable the development of more personalized and effective strategies for immunotherapy of cancer.

## 1. Introduction

Cancer immunotherapy represents a paradigm shift in cancer treatment by using the immune system to target and destroy cancerous cells [1,2]. In contrast to traditional therapies such as chemotherapy and radiotherapy, which tend to be non-specific, immunotherapy is more precise, augmenting the immune system’s ability to detect and destroy tumors [3,4]. Immune checkpoint inhibitors (ICIs), chimeric antigen receptor T-cell (CAR-T) therapy, and cancer vaccines continue to be among the most common methods used today [5,6]. Cancer ICIs, which include inhibitors of programmed cell death protein-1 (PD-1), programmed death-ligand 1 (PD-L1), and cytotoxic T-lymphocyte-associated antigen-4 (CTLA-4), work by releasing the brakes on T cells and thereby reinstating antitumor immunity [7]. Engineered T-cell therapy entails modifying a patient’s own T cells to express synthetic receptors that target specific tumor-associated antigens. This approach has been particularly effective in eliciting strong cytotoxic responses in blood cancers [8,9]. Parallel to this, the primary goal of cancer vaccines is to stimulate the immune system to recognize tumor antigens and enhance immune memory. However, the clinical efficacy of cancer vaccines against solid tumors has historically been poor [10,11]. The last decade has seen considerable success in the clinical applications of melanoma, non-small cell lung cancer, and some leukemias and lymphomas, driven by research and development in cancer immunotherapy [12,13]. Immunotherapy has the potential to transform the entire field of oncology because of durable responses and long-term remission achieved in subsets of patients. Nonetheless, the advantages are not universal [14,15]. A large number of patients do not respond or subsequently become resistant to treatment [16]. Significant obstacles that continue to limit treatment effectiveness include tumor heterogeneity, an immunosuppressive tumor microenvironment, and various mechanisms of immune evasion [17,18]. Some of this resistance is driven by tumor-intrinsic programs that remodel the microenvironment. In colon cancer, for example, NCOA3 blunts the response to anti-PD-L1 therapy through an HSP90α/EZH2/CXCL9 axis that limits T-cell recruitment [19]. JAK signaling is a more complicated case: loss of JAK function causes resistance, yet chronic JAK activity can also suppress the response to PD-1 blockade and may be targeted with JAK inhibitors [20]. In addition, immunotherapy has distinct toxicities, including immune-related adverse events (irAEs) in ICIs, and cytokine release syndrome (CRS) and neurotoxicity in CAR-T therapy, which need close observation and management [21,22]. Such limitations reaffirm the importance of reliable biomarkers in clinical decision-making for cancer immunotherapy [23,24]. Predictive biomarkers can help assess a patient’s potential response to a given treatment.

In contrast, prognostic biomarkers estimate a patient’s prognosis, and pharmacodynamic and safety biomarkers assess the efficacy and toxicity of a treatment [25,26]. Clinical practice incorporates PD-L1 expression and tumor mutational burden (TMB) to stratify patients and tailor treatment approaches, though their predictive value remains unclear [27,28]. Emerging biomarker research driven by rapid advancements in genomics, transcriptomics, and immune profiling is facilitating a more personalized approach to immunotherapy [27,28]. Tumor cell death pathways matter as well; PANX2, for instance, restrains lung adenocarcinoma by triggering disulfidptosis and enhancing antitumor immunity [29], an example of how tumor-intrinsic mechanisms connect to immune responses and, potentially, to new biomarkers.

### 1.1. Why Immunotherapy Fails in an Individual Patient: Prerequisites, Tumor Cell Death and Resistance

Every immunotherapy discussed in this review is approved for defined indications, so the practical problem is less whether a drug can work than why it does not work in a particular patient. Answering that question starts with pathophysiology. For a checkpoint inhibitor to act, a chain of conditions must be met: the tumor must carry antigens that T cells can see and then process them and present them on human leukocyte antigen (HLA) class I molecules; dendritic cells must prime tumor-specific T cells; those T cells must reach the tumor, recognize their targets and survive in the local environment; and the tumor cell must be susceptible to the killing signals that follow [30]. Cell therapies and bispecific engagers bypass some of these steps but depend on others, notably expression of the target antigen and the fitness and persistence of the effector cells. Each link in the chain can be measured: neoantigen load and TMB, HLA and B2M integrity, the inflamed, excluded or desert immune phenotype, interferon-γ (IFN-γ) signaling, and the suppressive cells and metabolites of the tumor microenvironment. Monitoring is most useful when it is aimed at the link most likely to have failed, and this logic guides the order of the sections that follow.

Immunotherapy ultimately works by killing tumor cells, and the mode of death matters for both efficacy and monitoring. Cytotoxic T cells and natural killer (NK) cells kill mainly through perforin and granzymes and through death receptors such as Fas, while the IFN-γ and tumor necrosis factor (TNF) they release act on tumor cells more widely. IFN-γ from CD8^+^ T cells also represses the cystine transporter SLC7A11 and so sensitizes tumor cells to ferroptosis [31]; granzyme A cleaves gasdermin B to trigger pyroptosis [32]; and granzyme B can activate gasdermin E, turning apoptosis into an inflammatory, pyroptotic death that further enhances antitumor immunity [33]. Cell death that exposes calreticulin and releases ATP and HMGB1, so-called immunogenic cell death, recruits and activates dendritic cells, and tumor DNA taken up by these cells stimulates type I interferon through the cGAS–STING pathway [34,35]. Other regulated forms of death, such as the disulfidptosis described above, are now being linked to antitumor immunity as well [29].

These mechanisms explain why additional treatments can enhance tumor cell death and immunotherapy response. Some chemotherapies, including anthracyclines and oxaliplatin, induce immunogenic cell death, with calreticulin exposure determining whether dying cells are immunogenic [36], and chemotherapy–ICI combinations have become a standard of care in non-small cell lung cancer (NSCLC) [37]. Radiotherapy releases tumor DNA that activates STING-dependent interferon signaling [35], and durvalumab given after chemoradiotherapy improved survival in stage III NSCLC [38]. Other strategies, including JAK inhibition to relieve chronic interferon signaling [39,40] and agents that promote ferroptosis or pyroptosis alongside checkpoint blockade [41], are at earlier stages of development. For monitoring, the implication is that markers of tumor cell death and immune activation, such as falling circulating tumor DNA (ctDNA), rising cytotoxic T-cell activity or imaging signs of immune infiltration, can be read as pharmacodynamic evidence that a combination is doing what it was intended to do (Section 9.2 and Section 9.5).

Resistance arises when one of these steps fails, either from the start or under treatment. Genetic routes are discussed in Section 3.5: loss of antigen presentation (B2M mutations, loss of HLA heterozygosity), defects in IFN-γ signaling (JAK1/JAK2) and oncogenic pathways that exclude T cells (STK11/KEAP1, WNT/β-catenin). Tumor stress responses are an important non-genetic route. Hypoxia induces PD-L1 directly through hypoxia-inducible factor-1α (HIF-1α) [42]; endoplasmic reticulum (ER) stress and activation of the unfolded protein response sensor XBP1 in tumor-associated dendritic cells impair their ability to present antigen [43]; autophagy in pancreatic cancer cells degrades MHC-I and so hides tumors from T cells [44]; and prolonged interferon signaling in tumor cells switches on a multigenic resistance program that includes several inhibitory ligands [45]. Stress responses therefore sit at the center of adaptive resistance, and many of them leave measurable traces, including hypoxia and interferon gene signatures, PD-L1 induction and changes in HLA expression. However, few have yet been tested as clinical biomarkers.

Whether drug activity can be monitored “online” and therapy individualized in real time is still only partly answered. Serial ctDNA measurements can show molecular response within weeks of starting treatment, repeated blood immune profiling can follow T-cell reinvigoration, and molecular imaging can visualize target engagement in the whole body (Section 9). Truly continuous monitoring of drug activity in patients is not yet possible, although the sensors and nanoprobes described in Section 9.5 are helping this field move in that direction. For now, treatment is individualized at defined decision points (selection before treatment, early response assessment, detection of resistance and adaptive changes in therapy), and the remainder of this review assesses how well current biomarkers support each of these decisions.

### 1.2. Scope and Evidence Framework of This Review

Biomarkers for immunotherapy have been reviewed many times, usually one therapy or one technology at a time. For clinicians and translational researchers, though, the more useful question is rarely whether a marker correlates with outcome; it is whether the marker is good enough to guide a treatment decision and, if not, what evidence is still missing. We have therefore organized this review around four criteria: analytical validity (whether the assay measures the analyte accurately and reproducibly), clinical validity (whether the result reliably separates patients by outcome), clinical utility (whether acting on the result improves outcomes or decisions) and regulatory readiness (whether there is an approved or guideline-endorsed assay with a defined cut-off and intended use) [46,47,48,49].

Tumor-intrinsic, microenvironmental, circulating and host-related (microbiome and metabolic) markers are discussed together here because they describe different layers of the same process, the cancer–immune set point [30]. Tumor-intrinsic features decide whether a tumor is antigenic and visible to T cells; the microenvironment decides whether effector cells arrive, function and reach the tumor; circulating markers report the systemic immune state and can be sampled repeatedly; and host factors influence all of these. Because response depends on several layers at once, no single marker performs well on its own, which is why composite approaches are attracting interest.

We address four questions. Which biomarkers are ready to guide treatment today, and in which therapy and cancer type? For promising markers that are not yet in routine use, what level of evidence is lacking? How do biomarker needs differ between checkpoint inhibitors, cell therapies, vaccines, bispecific engagers and oncolytic viruses? And what methodological work is required before markers derived from multi-omics or spatial profiling can be used in practice?

## 2. Classification of Biomarkers in Cancer Immunotherapy

Based on clinical application, biomarkers in cancer immunotherapy are classified into the following types: predicting the likelihood of a therapeutic response, estimating the course of a disease, measuring the effect of treatment, and determining the risk of treatment-related toxicity [50]. Optimizing patient selection, enhancing therapeutic effectiveness, and reducing negative side effects all benefit from this classification [51].

### 2.1. Predictive Biomarkers

Predictive biomarkers identify patients most likely to respond to a particular immunotherapeutic intervention [50]. These biomarkers help make treatment decisions by predicting the probability of a therapeutic response [25]. PD-L1 expression and TMB are two of the most researched predictive biomarkers [52]. Tumor/immune cell PD-L1 expression, most often evaluated by immunohistochemistry, has been linked to better response rates to immune checkpoint inhibitors targeting the PD-1/PD-L1 pathway [53]. Nonetheless, tumor heterogeneity, variation in scoring systems and differences between assay platforms can all reduce its predictive power [54]. TMB is a surrogate for the new antigen load, which improves the immunogenicity of the tumor, and it reflects the sum of somatic mutations in the tumor genome [55]. For melanoma and non-small cell lung cancer, high TMB is associated with better responses to checkpoint blockade therapies, confirming their potential usefulness [56]. However, challenges to their standardization and clinical applicability remain [57]. Microsatellite instability (MSI), deficiency in mismatch repair (dMMR), and certain gene expression profiles (associated with IFN signaling and immune activation) are other developing predictive biomarkers [58,59].

Several predictive markers are already part of routine care. PD-L1 testing with the 22C3 assay became a companion diagnostic after KEYNOTE-024, in which first-line pembrolizumab improved progression-free and overall survival over chemotherapy in NSCLC with a tumor proportion score (TPS) of at least 50% [60]. The combined positive score (CPS) now guides PD-1 blockade in head and neck squamous cell carcinoma (KEYNOTE-048), triple-negative breast cancer (KEYNOTE-355; CPS ≥ 10) and gastric or gastroesophageal junction adenocarcinoma (CheckMate 649) [61,62,63]. MSI-H/dMMR status, established by Le and colleagues [64,65], was the first tissue-agnostic biomarker for immunotherapy; its value was later confirmed by first-line pembrolizumab in MSI-H/dMMR colorectal cancer (KEYNOTE-177) [66] and by the high clinical complete response rate to dostarlimab in dMMR locally advanced rectal cancer [67]. High TMB (TMB-H; ≥10 mutations/megabase by FoundationOne CDx) led to tissue-agnostic FDA approval of pembrolizumab on the basis of the prospective biomarker analysis of KEYNOTE-158 [68,69]. Newer agents have added germline HLA type to this list: tebentafusp, a bispecific T-cell engager for metastatic uveal melanoma, requires HLA-A*02:01 [70], and the T-cell receptor (TCR)-engineered product afamitresgene autoleucel requires HLA-A*02 together with MAGE-A4 expression in synovial sarcoma [71].

### 2.2. Prognostic Biomarkers

Prognostic biomarkers inform clinicians regarding the likely course of a disease, independent of the treatment modalities available, i.e., the type of treatment administered [25]. Such biomarkers are useful for evaluating disease progression, the likelihood of patient survival, and the risk of disease recurrence [25]. Prognostic biomarkers in cancer immunotherapy include tumor staging, lactate dehydrogenase (LDH) levels, and tumor-infiltrating lymphocytes (TILs) [72]. Improved survival outcomes across several cancer types are associated with high levels of TILs, especially CD8^+^ cytotoxic T cells [73]. Moreover, systemic inflammatory markers, such as the neutrophil-to-lymphocyte ratio (NLR), indicate poor prognosis, suggesting an immunosuppressed systemic environment [74]. Although prognostic biomarkers may not predict response to immunotherapy, they play an important role in risk assessment and clinical decision-making [75].

A few prognostic markers have entered staging and pathology practice. Serum LDH forms part of the M category in the AJCC 8th edition melanoma staging system [76]. The Immunoscore, which uses digital pathology to count CD3^+^ and CD8^+^ T cells in the tumor core and invasive margin, was validated internationally in colon cancer [77,78], and stromal TILs in breast cancer are scored according to a standardized method from the International TILs Working Group [79]. In large pan-cancer cohorts, pretreatment NLR has also been linked to outcome after ICIs [80].

### 2.3. Pharmacodynamic Biomarkers

Pharmacodynamic biomarkers capture the biological response of a patient to an intervention and serve to evaluate the impact of the intervention and its mechanism of action in a time-sensitive manner [81]. Within the framework of immunotherapy, pharmacodynamic biomarkers include treatment-induced shifts in peripheral immune cell populations, variations in cytokines, and regulation of immune genes [82]. An example of immune activation and therapeutic response is increased immune checkpoint blockade in peripheral CD8^+^CD8^+^ T cells and increased levels of IFN-γ [83]. ctDNA serves as a pharmacodynamic biomarker that monitors tumor burden and detects response to/resistance from treatment during the course of the therapy. It can do so in a non-invasive manner [84]. With these biomarkers, it is possible to assess treatment response over an extended period and modify treatment strategies accordingly [25].

Well-documented examples include the early on-treatment proliferation (Ki-67^+^) of peripheral PD-1^+^ CD8^+^ T cells after anti-PD-1 therapy, which predicted response when expressed relative to tumor burden [85]; clonal expansion of intratumoral T cells during treatment [86]; and early falls in ctDNA, which tracked with longer survival in pan-cancer cohorts treated with pembrolizumab [87,88]. In hematological malignancies, measurable residual disease (MRD) already guides immunotherapy; blinatumomab, for instance, is approved for MRD-positive B-cell precursor acute lymphoblastic leukemia [89].

### 2.4. Safety Biomarkers

Safety biomarkers help predict or detect immune-related adverse events (irAEs), which are frequent side effects of cancer immunotherapy, especially immune checkpoint inhibitors and CAR-T cell therapy [25]. Immune-related adverse events result from overzealous immune activation and can involve multiple organs, including the integumentary, gastrointestinal, hepatic, and endocrine systems [90]. Research has focused on biomarkers, including autoantibody levels, cytokine levels such as interleukin-6 (IL-6), and immune subset cells, for predicting possible risks of toxicity [91]. In CAR-T cell therapy, rising levels of IL-6 and of the acute-phase protein ferritin accompany the development of CRS and neurotoxicity [92]; as discussed below, these are monitoring markers rather than validated pretreatment predictors [92]. Recognizing at-risk patients early enables clinicians to intervene and handle adverse consequences more efficiently [93].

In everyday practice, safety monitoring during ICI therapy rests on guideline-based laboratory tests at baseline and during treatment, including thyroid function, liver enzymes, glucose and cortisol [94]. For CAR-T cell therapy, Teachey and colleagues showed that cytokine profiles measured shortly after infusion (including IFN-γ, soluble gp130 and IL-1RA) predicted severe CRS [95]. IL-6 sits at the center of CRS biology, which is why the IL-6 receptor antagonist tocilizumab was approved for CAR-T-induced CRS [96]. CRS and immune effector cell-associated neurotoxicity syndrome (ICANS) are graded clinically with the ASTCT consensus criteria [97]. C-reactive protein (CRP) and ferritin are measured serially to follow inflammation, but they are non-specific and have not been validated as pretreatment predictors (Section 7.2). Markers of endothelial activation, such as the angiopoietin-2/angiopoietin-1 ratio and von Willebrand factor, are associated with severe neurotoxicity [98], and scores built from routine pre-infusion laboratory values, namely the modified EASIX and CAR-HEMATOTOX scores, have been validated retrospectively for CRS/ICANS and hematological toxicity, respectively [99,100].

### 2.5. Evidence Framework Used in This Review

Throughout this review, we place each biomarker in one of four tiers. Tier 1 (regulatory-approved/companion diagnostic) covers assays with a defined cut-off that a regulator such as the FDA or EMA has approved for selecting patients for a given therapy. Tier 2 (guideline-recommended) covers testing recommended by major guidelines (e.g., NCCN, ESMO, ASCO/CAP) or built into staging, whether or not it is a companion diagnostic. Tier 3 (clinically validated but not routinely used) covers markers whose association with outcome has been confirmed in independent cohorts or prospective trials but which still lack proven clinical utility, a standardized assay or a cut-off. Tier 4 (exploratory/preclinical) covers markers supported only by retrospective, single-cohort or preclinical data. Table 1 summarizes the tier assigned to each marker.

## 3. Tumor-Intrinsic Biomarkers

Tumor-intrinsic biomarkers stem from the genetic and molecular features of cancer cells and are critical in assessing tumor immunogenicity and the effectiveness of immunotherapy [101]. The analysis of these biomarkers has advanced our understanding of tumor–immune interactions, and their utility for patient stratification has been integrated into clinical practice [102].

### 3.1. PD-L1 Expression

Among the tumor-intrinsic biomarkers in cancer immunotherapy, PD-L1 expression has received the most extensive validation [103]. Within the tumor microenvironment, tumor cells and immune cells also express PD-L1, which suppresses the immune response and enables tumor immune evasion by binding to PD-1 receptors on T cells [104]. PD-L1 immunohistochemistry (IHC) results are also affected by pre-analytical factors such as tissue overfixation [105]. A variety of companion diagnostic assays have been developed, including 22C3, 28-8, and SP142, each of which employs different scoring methodologies, including the tumor proportion score (TPS) and the combined positive score (CPS) [106]. While there is considerable clinical experience, difficulties persist, including assay variability and a lack of standardization [107]. In various malignancies like melanoma and non-small cell lung carcinoma, elevated PD-L1 expression has been associated with increased responsiveness to PD-1/PD-L1 blockade [108]. Nonetheless, some responses have been observed in individuals with low or negative PD-L1 expression, suggesting low sensitivity and specificity [109]. Tumor heterogeneity, variable expression, and differences in testing platforms also limit its clinical applicability [110].

Part of the reason PD-L1 performs inconsistently lies in its biology. The amount of PD-L1 on a cell is not set by transcription alone; chromatin remodeling, transcriptional and post-transcriptional control, glycosylation, ubiquitination, membrane trafficking and exosomal release all contribute [111]. N-linked glycosylation stabilizes the protein [112] but can also hide the epitopes recognized by diagnostic antibodies; when tissue sections were deglycosylated, PD-L1 detection improved and correlated better with ICI response [113]. CMTM6 and CMTM4 keep PD-L1 at the cell surface by protecting it from lysosomal degradation [114,115], and the deubiquitinase CSN5 stabilizes it in response to inflammatory signals [116]. Exosomal PD-L1, which tissue IHC cannot detect, can suppress T cells at distant sites [117,118]. Expression is also inducible, because IFN-γ released by infiltrating T cells upregulates PD-L1 (adaptive immune resistance) [119]. Not surprisingly, PD-L1 varies across regions of the same tumor and between primary and metastatic lesions, so a small biopsy may misclassify a patient [120], and it can change over time with chemotherapy, radiotherapy or targeted therapy. Results also differ between platforms, which use different antibody clones, staining systems, scoring cells (tumor cells alone or tumor plus immune cells) and cut-offs; in the Blueprint project, 22C3, 28-8 and SP263 stained tumor cells similarly, whereas SP142 stained fewer [121,122]. These layers of regulation help explain why a single, static IHC result often fails to capture the real activity of the PD-1/PD-L1 axis, including the responses seen in PD-L1-negative tumors.

### 3.2. Tumor Mutational Burden

TMB is defined as the total number of somatic mutations per coding region of the tumor genome. It is an indirect measure of the tumor’s ability to generate an immune response [123]. An increased TMB increases the likelihood of generating neoantigens. Neoantigens are new peptide sequences that MHC molecules present, and the immune system recognizes them as new and foreign [55]. This will augment T-cell activation and also improve sensitivity to immune checkpoint inhibitors [124]. TMB has emerged as a predictive biomarker of immune response, with higher TMB correlating with more favorable clinical outcomes in the treatment of cancer, including melanoma and lung cancer [125]. The U.S. FDA has approved pembrolizumab for use against any tumor as long as it has a high TMB (≥10 mutations/megabase) [69]. However, concerns regarding these tests include variability of sequencing platforms, absence of a standardized threshold, high costs, and inconsistent predictive value across various cancers [126].

### 3.3. Microsatellite Instability and Mismatch Repair Deficiency

Defects in the DNA mismatch repair system cause MSI and dMMR, leading to the accumulation of insertion–deletion mutations in microsatellite regions [58]. MSI-high (MSI-H) and dMMR status are among the first tumor-agnostic biomarkers to receive U.S. FDA approval for immunotherapy [127]. Pembrolizumab has been approved for use in patients with unresectable or metastatic MSI-H/dMMR solid tumors, representing a significant milestone in the development of biomarker-based, tissue-agnostic therapies [128]. The MSI-H/dMMR tumors have high mutation rates and generate neoantigens, leading to increased immune cell infiltration and greater responsiveness to checkpoint inhibitors [129]. This particular biomarker is especially important when it comes to colorectal, endometrial, and gastric cancers, and is also seen across many other tumor types [130].

### 3.4. Neoantigens and Other Genomic Alterations

Neoantigens, derived from somatic mutations, are specific to tumors and are fundamental to cancer’s immunological properties [131]. Neoantigens are displayed on the surfaces of cancer cells using MHC molecules and are detected by T cells as foreign entities, thereby initiating an immune response [132]. The quantity and relative quality of neoantigens are primary determinants of response to immunotherapies, particularly checkpoint inhibitors [133]. Next-generation sequencing (NGS) and computational biology advancements made it possible to discover personalized neoantigens, which opened new horizons for custom immunotherapeutic strategies, including neoantigen vaccines and adoptive T-cell therapies [134]. Additional specific genomic alterations, such as immune-modulatory mutations in POLE, KRAS, and TP53, offer prospects as biomarkers for stratified interventions [135].

### 3.5. Resistance-Associated Tumor-Intrinsic Alterations

Several tumor-intrinsic alterations are linked to primary or acquired resistance to ICIs, and loss of antigen presentation is the best described. Loss-of-function mutations or loss of heterozygosity in B2M, the gene encoding β2-microglobulin, abolish surface HLA class I and have been found in melanomas and lung cancers that became resistant to PD-1 blockade [136,137,138]. Allele-specific loss of HLA heterozygosity (HLA LOH), which is common in NSCLC, narrows the range of neoantigens that can be presented [139], and germline homozygosity at HLA class I loci has been associated with shorter survival after ICIs [140]. Inactivating JAK1 or JAK2 mutations make tumor cells deaf to IFN-γ, preventing IFN-induced PD-L1 and MHC expression, and have been implicated in both acquired and primary resistance [136,141]. The JAK pathway cuts both ways, however: chronic IFN/JAK signaling can itself drive resistance, and early-phase trials in NSCLC and Hodgkin lymphoma found better responses when JAK inhibitors were added to PD-1 blockade [20,39,40]. Oncogenic signaling can also keep T cells out. In KRAS-mutant lung adenocarcinoma, co-mutation of STK11/LKB1 or KEAP1 is associated with a “cold” microenvironment and poorer outcomes on PD-(L)1 inhibitors [142,143]; tumor-intrinsic WNT/β-catenin activation blocks the recruitment of CD103^+^ dendritic cells and T cells in melanoma [144]; and PTEN loss promotes resistance to T-cell-mediated therapy [145]. Among driver alterations, EGFR mutations and ALK rearrangements in NSCLC predict low response rates to PD-1 pathway blockade [146]. More recent mechanistic work has added NCOA3, which impairs anti-PD-L1 efficacy in colon cancer through an HSP90α/EZH2/CXCL9 axis [19], and PANX2, whose induction of disulfidptosis enhances antitumor immunity in lung adenocarcinoma [29]. Apart from EGFR/ALK status, which already steers NSCLC patients toward targeted therapy rather than ICIs, these alterations remain Tier 3–4 markers (Table 1); whether they are truly predictive rather than prognostic, and whether they should ever be used to withhold treatment, has yet to be tested prospectively.

## 4. Tumor Microenvironment (TME)-Based Biomarkers

The TME comprises a variety of tumor cells, immune cells, stroma, and other soluble components, forming a complex and dynamic system [147]. The TME is essential for reconfiguring anti-tumor immune responses and is a major determinant of the efficacy of cancer immunotherapy [148]. The tumor microenvironment generates biomarkers that indicate immunostimulation, immunosuppression, and potential therapeutic response [149].

### 4.1. Tumor-Infiltrating Lymphocytes (TILs)

TILs are a crucial part of the immune system’s response to the tumor [150]. The direct involvement of CD8^+^ cytotoxic T cells in recognizing and eliminating tumor cells makes them indispensable in this cohort [151]. Importantly, tumors with high CD8^+^ T cell density are likely to have an “increased or hot” tumor phenotype and therefore respond to immune checkpoint inhibitors [152]. CD8^+^ T cells can drive antitumor immune responses via the release of proteolytic factors—perforin and granzyme B—and espouse inflammatory responses via the release of IFN-γ [153]. Multiple studies have shown improved clinical outcomes and survival in various cancers with increased infiltration of CD8^+^ T cells [73]. Nevertheless, not every TIL is functionally active. Exhausted or dysfunctional T cells can express inhibitory receptors, including PD-1 and TIM-3, which impair their capacity to mount an antitumor response [154]. As a result, TILs, both in number and function, are determinants of therapeutic response [155].

### 4.2. Immune Gene Expression Signatures

Using gene expression profiling, researchers have documented immune-centric transcriptional signatures that correlate with the tumor microenvironment’s immune status [156]. One of the most documented biomarkers linked to an individual’s response to immunotherapy is the IFN-γ gene signature [157]. These signatures comprise genes related to antigen presentation, T-cell activation, and cytotoxic effector functions, including CXCL9, CXCL10, IDO1, and HLA genes [158]. Tumors that demonstrate a strong IFN-γ signature tend to have robust immune cell infiltration and are more likely to respond to PD-1/PD-L1 blockade therapies [159]. On the other hand, tumors lacking these characteristics are generally identified as “cold” tumors, with little to no immune infiltration and a poor response to immune therapeutic strategies [160]. Although the integration of gene panels offers future potential, several issues remain regarding consistency and their incorporation into everyday clinical practice [161].

### 4.3. Cytokines and Chemokines

Cytokines and chemokines are soluble mediators involved in the recruitment, activation, and signaling of immune cells in the TME [162]. Some pro-inflammatory cytokines, such as IFN-γ, interleukin-2 (IL-2), and tumor necrosis factor-α (TNF-α), promote antitumor immune responses by increasing T-cell activation and cytotoxicity [163]. In contrast, IL-10 and transforming growth factor-β (TGF-β) are immunosuppressive cytokines that inhibit immune responses and promote tumor progression [164]. On the other hand, chemokines such as CXCL9 and CXCL10 are instrumental in recruiting effector T cells into the tumor microenvironment, thereby improving the efficacy of immunotherapy [165]. Cytokines and chemokines have been shown to predict treatment response and toxicity, making them useful as both predictive and pharmacodynamic biomarkers [166]. However, due to their constantly changing nature, clinical applications are more complicated [167].

### 4.4. Immune Suppressive Cells

Immunosuppressive cell populations in the TME constitute a primary obstacle to tumor-targeting immunity and are critical contributors to the inability to respond to immunotherapy [168]. Regulatory T cells (Tregs), myeloid-derived suppressor cells (MDSCs), and tumor-associated macrophages (TAMs) form the immunosuppressive components of the TME. Tregs suppress effector T-cell functions via the secretion of immunosuppressive cytokines (e.g., IL-10, TGF-β) and through direct cell–cell contact [169]. MDSCs inhibit T-cell proliferation and function through mechanisms such as arginase production and the generation of reactive oxygen species [170]. TAMs, especially the M2-polarized subtype, promote tumor development, angiogenesis, and immune suppression [171]. Prognosis and responses to immunotherapy are generally unfavorable for those with high infiltration of these suppressive cells [172]. While the presence of the above-mentioned immunosuppressive cell types is a potential barrier to the development of effective immunotherapy treatments, they may also serve as important biomarkers of therapeutic resistance [149].

### 4.5. Tertiary Lymphoid Structures

Tertiary lymphoid structures (TLS) are ectopic lymphoid aggregates, with B-cell follicles, T-cell zones and dendritic cells, that develop in chronically inflamed tissues, including tumors. In 2020, three studies linked TLS and B-cell signatures to ICI response in melanoma, renal cell carcinoma and soft-tissue sarcoma [173,174,175]. The PEMBROSARC trial then provided prospective evidence: restricting enrolment to TLS-positive sarcomas raised the objective response rate to pembrolizumab compared with unselected cohorts [176]. TLS are thus one of the few TME markers tested in a prospective, biomarker-enriched design (Tier 3), although definitions of TLS maturity and scoring methods still vary.

### 4.6. Spatial Immune Phenotypes

Where T cells sit matters as much as how many there are. Tumors are commonly described as immune-inflamed (T cells in contact with tumor cells), immune-excluded (T cells held in the stroma) or immune-desert (few T cells) [30,177]. In urothelial cancer, the excluded phenotype was associated with TGF-β signaling in fibroblasts and with lack of response to atezolizumab [178]. In contrast, in melanoma the density of CD8^+^ T cells at the invasive margin predicted response to pembrolizumab [119]. The internationally validated Immunoscore shows the prognostic value of combining cell type, density and location [78].

### 4.7. T-Cell Receptor Clonality

TCR sequencing measures the diversity and clonality of T-cell populations. Higher intratumoral TCR clonality at baseline was associated with response to pembrolizumab [119], and anti-PD-1 therapy expands tumor-reactive clones [86]. Paired single-cell RNA and TCR sequencing later showed that responding clones are often newly recruited rather than reinvigorated pre-existing clones [179], an argument for following peripheral TCR repertoires during treatment. Because assays and analysis pipelines are not standardized, TCR metrics remain in Tier 4.

### 4.8. Multiplex Imaging and Spatial Transcriptomics

Multiplex immunohistochemistry/immunofluorescence (mIHC/IF) and imaging mass cytometry quantify several markers (for example, CD8, PD-1, PD-L1, FOXP3, CD68 and pan-cytokeratin) on a single section while preserving spatial information. In a meta-analysis of more than 8000 patients, mIHC/IF predicted response to anti-PD-1/PD-L1 therapy with a higher pooled area under the curve (AUC 0.79) than PD-L1 IHC, TMB or gene expression profiling alone [180]. Imaging mass cytometry and single-cell spatial atlases have since identified chemokine niches and cellular neighborhoods associated with ICI response and outcome [181,182], and spatial transcriptomics, which adds genome-wide expression in tissue context, has mapped risk-associated cell types and regulatory programs as colorectal cancer progresses [183]. These approaches are still classified as Tier 4; moving them forward will require analytically validated panels, harmonized image analysis and spatial metrics defined in advance and tested in independent cohorts.

Taken together, stromal TIL assessment and the Immunoscore are clinically validated prognostic tools, and TLS have been evaluated in a prospective biomarker-enriched cohort (Tier 3). IFN-γ-related gene expression profiles are clinically validated but not routinely used, while cytokine and chemokine levels, suppressive cell populations, TCR metrics and spatial metrics remain exploratory (Tier 4; Table 1).

## 5. Circulating and Non-Invasive Biomarkers

Liquid biopsies, including circulating and non-invasive biomarkers, have gained traction in cancer immunotherapy research, given their unique potential to provide real-time, evolving perspectives on tumors and their immune interactions [184]. Compared to tissue-based biomarkers, these methods offer greater flexibility for measuring tumor heterogeneity, owing to their longitudinal, less invasive, and heterogeneous tumor profile monitoring attributes [185]. ctDNA consists of fragmented DNA released into the circulation through the apoptosis or necrosis of tumor cells [186]. It is an extremely sensitive biomolecular indicator of tumor burden or molecular characterization [187].

ctDNA is commonly used to detect MRD after treatment and to detect relapse before radiographic progression [188]. In immunotherapy, ctDNA levels before, during, and after treatment may indicate how the therapy is working. Levels that decrease indicate the therapy is working; levels that increase indicate the therapy is not working, the disease is worsening, or the patient is becoming resistant to therapy [189]. Furthermore, ctDNA analysis enables the detection of specific tumor mutations and neoantigens, thereby aiding the development of personalized therapeutic approaches [190].

**Figure 1:**
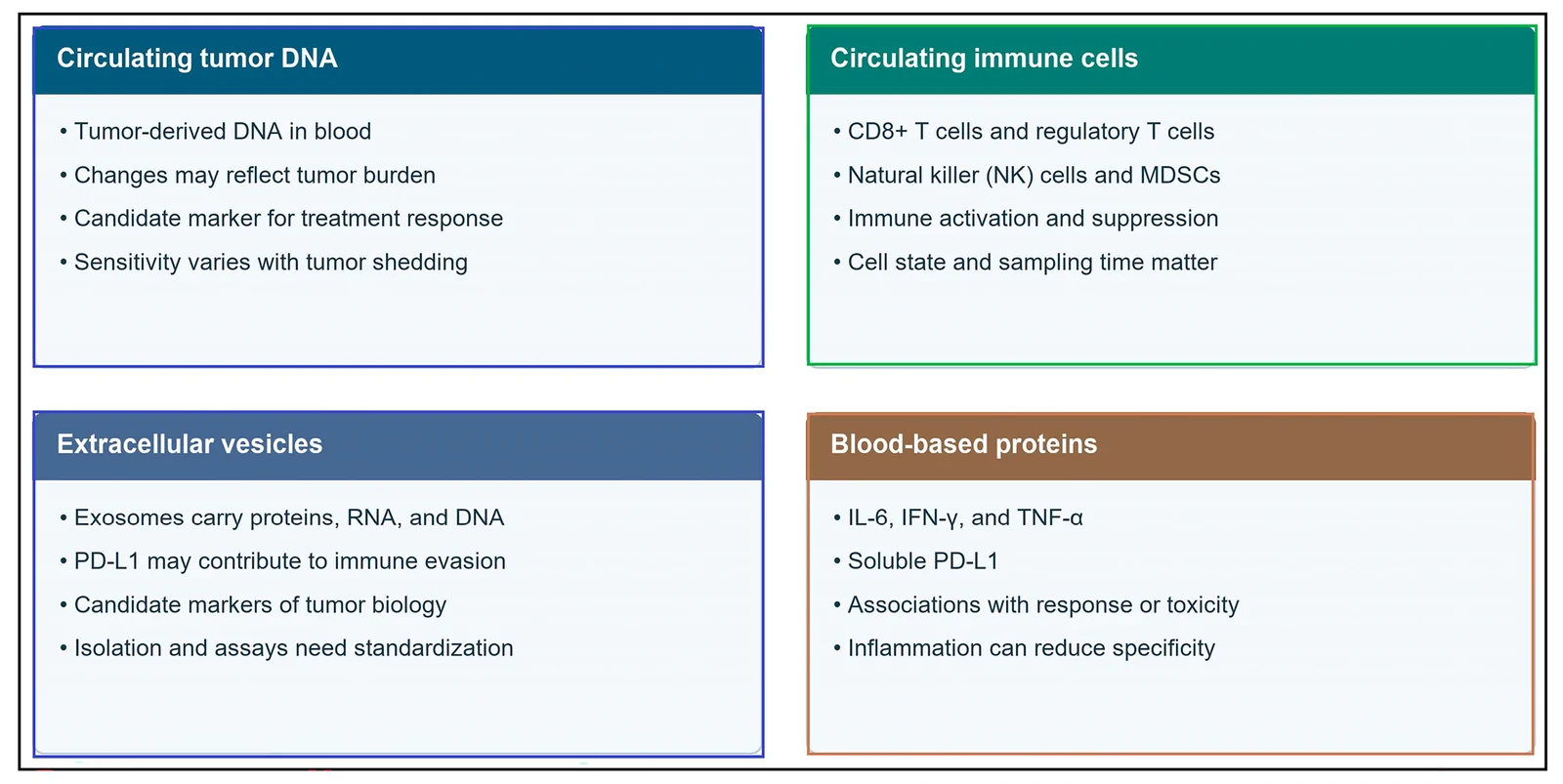
Circulating and non-invasive biomarkers in cancer immunotherapy. The four panels summarize the main classes of blood-based biomarkers discussed in Section 5: circulating tumor DNA (ctDNA), whose levels may reflect tumor burden and treatment response but depend on tumor shedding; circulating immune cells (CD8^+^ T cells, regulatory T cells, natural killer cells and myeloid-derived suppressor cells), which reflect systemic immune activation and suppression; extracellular vesicles, which carry proteins (including PD-L1), RNA and DNA; and blood-based proteins (IL-6, IFN-γ, TNF-α and soluble PD-L1), which are associated with response or toxicity but have limited specificity. Longitudinal measurements may complement tissue biomarkers, but prospective validation is required before biomarker-guided treatment changes.

Moreover, challenges such as limited availability of early-stage disease, assay sensitivity, and a lack of standardization remain barriers to widespread clinical use [191]. Systemic immune profiling and the evaluation of immunotherapy potential involve various analyses of immune cells in peripheral blood [192]. Circulating immune cell subtypes, including CD8^+^ T cells, regulatory T cells, natural killer cells, and myeloid-derived suppressor cells, contribute to understanding immune activation and suppression [193]. Following immune checkpoint blockade, enhanced clinical responses have been observed, accompanied by the activation and proliferation of peripheral CD8^+^ T cells [194]. Conversely, adverse therapeutic outcomes correlate with the increased presence of certain immunosuppressive cell types, including Tregs and MDSCs [195]. Peripheral blood profiling, despite its variability and lack of standardized assays, offers a flexible, reproducible method for capturing the nuances of the immune response throughout the course of therapy [185]. Exosomes and other extracellular vesicles (EVs) secreted by tumors and immune cells are membrane-bound entities that contain proteins, lipids, and nucleic acids characteristic of the parent cell [196]. Exosomes derived from tumors play an essential role in cell-to-cell communication within the tumor microenvironment and can modulate immune responses [197]. They can express immune checkpoint proteins, such as PD-L1, potentially leading to systemic immune evasion and resistance to immunotherapy [198]. Exosomal RNA and DNA have potential utility in tumor mutation profiling and assessment of treatment response [199]. Exosomes’ circulation stability and tumor heterogeneity encapsulation provide evidence for their potential as a non-invasive biomarker [200]. The clinical applicability of these procedures is undermined by the complexities associated with isolation, characterization, and standardization [201]. Protein biomarkers found in the bloodstream, such as cytokines, chemokines, and soluble immune checkpoint proteins, provide valuable information about the systemic immune response and the level of inflammation [201]. Cytokines, such as IL-6, IFN-γ, and TNF-α, play regulatory roles in immune responses and have been correlated with both clinical response and adverse events [202]. Increased levels of IL-6, for example, are correlated with CRS in patients who have received CAR-T cell therapy [203]. However, IL-6 is a non-specific marker used for monitoring rather than a validated pretreatment predictor (Section 7.2) [203]. Soluble PD-L1 (sPD-L1) in plasma has been studied as a potential biomarker for evaluating clinical response to immune checkpoint inhibitors [204]. Elevated levels of sPD-L1 are linked to a negative prognosis and to a decrease in the effectiveness of treatment [205]. Though protein biomarkers in blood are relatively inexpensive and easier to obtain, their clinical applicability is diminished by biological variability, nonspecificity, and the presence of non-tumor-associated influences [206] (Figure 1).

Of the circulating markers, tumor-informed ctDNA is the most mature: it has been clinically validated for MRD detection and early response assessment, and prospective ctDNA-guided trials are under way (Tier 3). Peripheral immune-cell profiling, extracellular vesicles, soluble PD-L1 and serum cytokines are still exploratory (Tier 4), and IL-6, CRP and ferritin, although used clinically to monitor CRS, have not been validated as predictors (Table 1).

## 6. Microbiome and Metabolic Biomarkers

New data suggest that host-related factors, specifically the gut microbiome and the host’s metabolic environment, may influence the mechanisms of antitumor immunity and the effects on immunotherapy for cancer [207]. These biomarkers, with a more systematic approach that goes beyond tumor-intrinsic and microenvironmental changes, aid in identifying novel shifts in therapies and in the development of customized treatment strategies [208]. The microbiota consists of trillions of microorganisms that inhabit the entire gastrointestinal tract. The microbiota also helps maintain immune homeostasis and regulate systemic inflammation [209]. The latest studies indicate that the composition and diversity of the gut microbiota impact how patients respond to immune checkpoint inhibitors [210]. Responses to anti-PD-1/PD-L1 therapies show positive correlations with particular bacterial taxa including *Akkermansia muciniphila*, *Bifidobacterium* spp. and *Faecalibacterium* [211]. These microorganisms enhance antigen presentation, facilitate dendritic cell maturation, and activate CD8^+^ T cells, thereby strengthening the immune response to tumors [212]. Conversely, the decreased microbial diversity, or dysbiosis, has been linked to unfavorable clinical outcomes and resistance to immunotherapy [213].

Moreover, antibiotics that disturb gut microbiota have been shown to diminish the efficacy of immunotherapy [214]. Analyses are being conducted on methods such as fecal microbiota transplantation (FMT), probiotics, and dietary alterations to assess how these methods can alter the microbiome and improve treatment response [215]. Nonetheless, obstacles such as differences among individuals and the lack of uniform methodologies continue to pose substantial difficulties [216]. Metabolite analysis examines the spectrum of small metabolites in biological systems and provides insight into the metabolic status of both tumor and immune cells [217]. The role of metabolic pathways in modulating immune cell activity and advancing tumors is crucial [218]. Typically, cancer cells undergo metabolic reprogramming characterized by increased glycolysis (also known as the Warburg effect). This increase in glycolysis creates a hostile microenvironment that impairs immune cell function [219]. Certain metabolites, such as lactate, kynurenine, and adenosine, exert immunosuppressive effects and are associated with reduced immunotherapy efficacy [220]. For example, high levels of lactate can inhibit T-cell proliferation and cytokine production, and kynurenine, produced by the enzymatic activity of indoleamine 2,3-dioxygenase (IDO), drives T-cell exhaustion [221].

**Figure 2:**
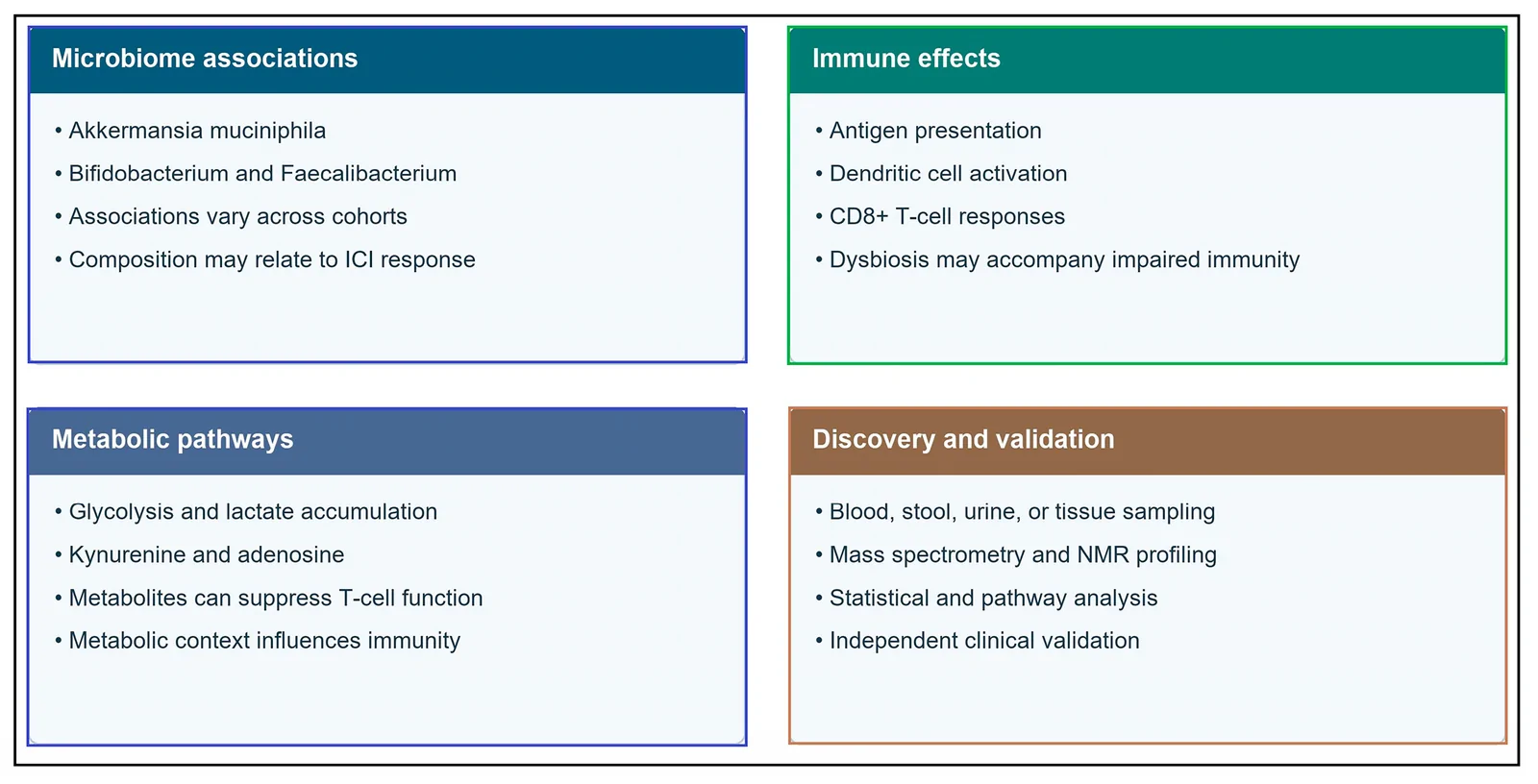
Microbiome and metabolic biomarkers in cancer immunotherapy. Gut bacterial taxa such as *Akkermansia muciniphila*, *Bifidobacterium* and *Faecalibacterium* have been associated with response to immune checkpoint inhibitors, although associations vary across cohorts. Proposed immune effects include enhanced antigen presentation, dendritic cell activation and CD8^+^ T-cell responses, whereas dysbiosis may accompany impaired immunity. Tumor metabolic pathways, including glycolysis with lactate accumulation and the production of kynurenine and adenosine, can suppress T-cell function. Discovery and validation of these biomarkers involve sampling of blood, stool, urine or tissue; profiling by mass spectrometry or nuclear magnetic resonance (NMR); statistical and pathway analysis; and independent clinical validation. Antibiotics may alter microbial composition; fecal microbiota transplantation, probiotics and dietary interventions remain investigational.

On the other hand, immune cell activation and function can be enhanced by the presence of supportive metabolic profiles and increased availability of the relevant amino acids [222]. Predicting the response to immunotherapy and determining new targets for therapy can be achieved through metabolomic profiling of blood and tumor samples [223]. Metabolomics presents promise for potential integration in clinical practice; however, it remains in early phases due to a number of obstacles, including, but not limited to, analytical and systemic complexity, and integration with other biomarker systems [224] (Figure 2).

For now, microbiome composition and metabolite profiles remain exploratory (Tier 4). Associations differ from cohort to cohort and no microbial or metabolic signature has been validated prospectively for patient selection; the most clinically actionable microbiome-related factor at present is recent antibiotic exposure (Table 1).

## 7. Biomarkers for Specific Immunotherapies

To fully understand the challenges in predicting patient response and associated toxicity risks, and to improve patient selection for specific cancer treatment modalities, there is a need to examine treatment-specific biomarkers in immuno-oncology [225]. Immune checkpoint inhibitors, CAR-T cell therapies, cancer vaccines, and other immunotherapeutic strategies function through different mechanisms of action and are associated with different biomarker signatures [226].

### 7.1. Immune Checkpoint Inhibitors

The reestablishment of T-cell-mediated antitumor immunity has been achieved through ICIs targeting PD-1, PD-L1, and CTLA-4, thereby changing the landscape of cancer therapy [7]. When assessing potential response to anti-PD-1/PD-L1 therapies, evaluating PD-L1 expression on tumor cells or immune cells is among the most common approaches, despite its limitations [227]. PD-L1 expression that elicits a stronger response is common, although the predictive value of the biomarker is diminished by varying methods of tumor detection and tumor heterogeneity [228]. TMB and MSI are key biomarkers for ICIs, since they correlate with tumor immunogenicity and neoantigen expression levels [229].

Furthermore, immune-related gene expression profiles, especially those related to IFN-γ signaling and expression, have been correlated with positive outcomes to checkpoint blockade therapy [159]. Unlike other immune checkpoint inhibitor classes, CTLA-4 inhibitors have fewer well-established biomarkers but have shown promising predictive capabilities for immune activation status, T-cell repertoire diversity, and TIL density [230]. Although progress has been made, no single biomarker demonstrates adequate sensitivity and specificity, leading to the increasing study of composite biomarker strategies [231]. The evidence tier of each ICI biomarker is summarized in Table 1 [231].

### 7.2. CAR-T and CAR-NK Cell Therapy

CAR-T cells are engineered to recognize tumor-associated antigens and are best established in hematological malignancies [232], where biomarkers are sought for both efficacy and toxicity [233,234]. These markers fall into six biologically distinct groups. The first is target antigen density and antigen loss. CAR-T activity requires a threshold density of target antigen [235], and relapse with CD19-negative or CD19-low disease, caused for example by CD19 mutations or alternative splicing, is a major route of escape [236], so antigen status should be rechecked at relapse. The second is product phenotype: infusion products rich in memory-like CD27^+^CD45RO^−^CD8^+^ T cells were associated with durable remission in chronic lymphocytic leukemia [237], whereas products dominated by exhausted (e.g., LAG-3^+^, TIM-3^+^) CD8^+^ T cells were associated with poor response in large B-cell lymphoma [238]. The third is the fitness of the starting T cells; the composition and differentiation state of the apheresis product, often shaped by prior therapy, influence both manufacturing success and product quality [237]. The fourth is in vivo expansion and persistence. Peak CAR transgene levels and the area under the expansion curve, measured by qPCR or flow cytometry, correlate with response [237,239]; durable responses also go with activation and clonotypic expansion of the patient’s native cytotoxic T cells [240]; and in acute lymphoblastic leukemia, loss of persistence or B-cell recovery may herald CD19-positive relapse. The fifth is tumor burden and host inflammation, since a high baseline tumor burden and pretreatment systemic inflammation (for example, elevated LDH and IL-6) are associated with fewer durable responses and more toxicity [239,241,242]. The sixth comprises inflammatory and endothelial toxicity markers: cytokine profiles measured soon after infusion predicted severe CRS [95], endothelial activation (a raised angiopoietin-2/angiopoietin-1 ratio and von Willebrand factor) is associated with ICANS [98,243], and the modified EASIX and CAR-HEMATOTOX scores have been validated retrospectively [99,100].

IL-6, CRP and ferritin deserve specific caution. They are non-specific inflammatory markers whose main use is to follow the course of CRS and hemophagocytic lymphohistiocytosis (HLH)-like toxicities after infusion, where their kinetics help clinical management. They also rise with infection and other inflammatory states [244] and have not been validated as pretreatment predictors of CRS or efficacy; a raised baseline value mostly reflects tumor burden and systemic inflammation rather than a specific CAR-T-related risk. Cord blood-derived CAR-NK cells produced responses without major CRS or neurotoxicity in an early trial in CD19-positive lymphoid tumors [245], but biomarker data for CAR-NK therapy are still sparse [232,240,244].

### 7.3. TCR-Engineered T-Cell Therapy

TCRs recognize peptides presented by HLA, so TCR-engineered T-cell (TCR-T) therapies depend on two biomarkers: the restricting HLA allele and expression of the target antigen. Afamitresgene autoleucel, for example, requires HLA-A*02 and MAGE-A4 [71], and loss of HLA class I or of the target antigen (Section 3.5) offers the tumor an obvious route of escape.

### 7.4. Tumor-Infiltrating Lymphocyte Therapy

Adoptive cell therapies boost tumor-specific immunity by transferring immune effector cells directly [246]. Lifileucel was approved by the FDA in 2024 for advanced melanoma that had progressed on anti-PD-1 therapy [247], and in a randomized phase 3 trial TIL therapy prolonged progression-free survival compared with ipilimumab [248]. The quality, breadth and durability of the infused T cells are important determinants of outcome [249]. Candidate markers include the number of infused CD8^+^ T cells, the proportion of tumor-reactive and neoantigen-specific clones, and the presence of stem-like CD39^−^CD69^−^ CD8^+^ T cells, which have been associated with complete response and persistence [72,250]; none of these have yet been used to select patients.

### 7.5. Therapeutic Cancer Vaccines

Vaccines aim to stimulate tumor-specific immunity, and their main pharmacodynamic readouts are signs of immune activation such as expansion of tumor-specific T cells, increased IFN-γ secretion and enhanced antigen presentation [149,251]. Pre-existing immunity and strong T-cell responses against tumor-associated antigens or neoantigens are central to their activity [252]. For the approved cellular vaccine sipuleucel-T, there is no predictive biomarker [253]. Personalized neoantigen vaccines rely on genomic and transcriptomic profiling to identify and rank candidate neoantigens [254], and the vaccine-induced T-cell response is the key pharmacodynamic marker: in pancreatic cancer, vaccine-expanded neoantigen-specific T cells were associated with delayed recurrence [255], and in resected melanoma an mRNA neoantigen vaccine plus pembrolizumab improved recurrence-free survival [256]. These responses are measured with research assays such as ELISpot, peptide–MHC multimers and TCR tracking, and differences in immune status between patients, together with tumor heterogeneity, still make validation difficult [102].

### 7.6. Bispecific Immune Cell Engagers

For bispecific engagers, the relevant markers are target expression, HLA type for TCR-based molecules, and disease burden. Tebentafusp requires HLA-A*02:01 [70], and an early fall in ctDNA during treatment was associated with overall survival [257]. Blinatumomab is approved for MRD-positive B-cell precursor acute lymphoblastic leukemia [89], whereas the DLL3-targeted engager tarlatamab is active in small cell lung cancer without selection by DLL3 expression [258]. CRS monitoring with cytokines follows the same principles and carries the same limitations as in CAR-T therapy.

### 7.7. Oncolytic Viruses

Talimogene laherparepvec is approved for unresectable melanoma [259]. Oncolytic virotherapy increases intratumoral CD8^+^ T-cell infiltration and IFN-γ gene expression and can sensitize tumors to anti-PD-1 therapy, which makes on-treatment immune infiltration a candidate pharmacodynamic marker [260]; no validated predictive biomarker exists for this class.

Across these modalities, the markers used to select patients are those that define the drug target or its restricting element: PD-L1, MSI-H/dMMR, TMB-H, HLA type, MAGE-A4, CD19/BCMA and MRD. Markers of response, durability and toxicity for cell therapies, vaccines, bispecific engagers and oncolytic viruses are still mostly at Tier 3–4 (Table 1) [102,149,246,249,252,254].

## 8. Emerging Technologies in Biomarker Discovery

Biomarker discovery potential in cancer immunotherapy has recently been transformed by new high-throughput technologies and computational tool development [261]. Such new methods facilitate a novel understanding of tumor biology, the immune response, and patient heterogeneity, enabling precision oncology and personalized therapy [262].

New technologies change practice only when a discovery can be reduced to a simple, analytically validated assay and confirmed in independent patients. Genomics shows how this works. Exome-level observations linking mutational load and mismatch repair deficiency to ICI response were translated into targeted NGS panels and MMR IHC, which are now Tier 1 markers (TMB-H, MSI-H/dMMR). Transcriptomics produced the T-cell-inflamed gene expression profile, developed on a standardized RNA platform and validated across KEYNOTE trials [263,264]; it has not been adopted clinically, largely because it adds little to PD-L1 and TMB (Tier 3). Single-cell RNA sequencing identified TCF7^+^ memory-like CD8^+^ T cells as a correlate of ICI response in melanoma, and the finding was then turned into an immunofluorescence assay and confirmed in an independent cohort [265], a useful model for how single-cell discoveries can become practical tests. Spatial profiling has progressed furthest through simple readouts such as TLS scoring, tested prospectively in PEMBROSARC [176], and mass cytometry of blood showed that the pretreatment frequency of classical monocytes predicted response to anti-PD-1 therapy, a result reproduced by conventional flow cytometry in a separate cohort [266].

### 8.1. Multi-Omics Integration

Multi-omics involves integrating multiple molecular data types, such as genomics, transcriptomics, proteomics, epigenomics, and metabolomics, to provide an in-depth understanding of the interplay between the tumor and the immune system [267]. Genomic analyses reveal somatic mutations, copy number changes, and neoantigens, which aid in predicting tumor immunogenicity and the tumor’s response to immune checkpoint blockade [55]. Transcriptomic profiling highlights patterns of expression of immune-related genes, including pathways involved in interferon-γ expression, which are indicative of a therapeutic response [268]. Proteomics encompasses the measurement of proteins, their expression, and the various forms of post-translational modification. It has elucidated mechanisms of immune signaling and led to the discovery of soluble immune biomarkers, such as cytokines and immune checkpoint proteins [269]. These datasets enable multi-omics to gain a holistic understanding of tumor–immune interactions and facilitate the identification of novel predictive, prognostic, and pharmacodynamic biomarkers [270].

### 8.2. Single-Cell and Spatial Technologies

Technologies for single-cell sequencing enable the identification of distinct cells within tumors and peripheral blood. Unlike bulk-cell methods, this approach provides higher resolution for analyzing heterogeneity at the single-cell level.

Additionally, single-cell RNA sequencing (scRNA-seq) enables parsing of the tumor microenvironment and detection of distinct T cell subpopulations, including exhausted T cells, regulatory T cells, and myeloid suppressor cells [271]. At the same time, clonal expansion and treatment response diversity can be measured through parallel single-cell TCR sequencing [272]. This technology helps in understanding resistance mechanisms, predicting responses to immunotherapy, and tailoring treatment strategies [273]. In addition, multiparametric single-cell proteomics and spatial transcriptomics deepen our understanding of cell interactions and tissue architecture, paving the way for additional biomarker discovery [274].

Single-cell RNA sequencing resolves cell states that bulk profiling averages away, such as stem-like (TCF7^+^) versus terminally exhausted CD8^+^ T cells, regulatory T cells and suppressive myeloid populations, and relates them to treatment response [265,275]. In colorectal cancer, single-cell and spatial analyses across disease stages have identified cell types and regulatory programs associated with clinical risk [183], and single-cell profiling of the systemic immune “macroenvironment” has shown dysfunction and plasticity of immune cells well beyond the tumor [276]. Studies of this kind can nominate cell type-specific markers that are then measured with simpler, clinically feasible assays such as IHC, flow cytometry or targeted RNA panels.

### 8.3. Multi-Biomarker Panels and Predictive Clinical Models

Since any single marker captures only part of the cancer–immune interaction, combining markers tends to improve prediction. Across KEYNOTE trials, TMB and the T-cell-inflamed gene expression profile were independently predictive and, used together, separated groups with clearly different response rates [264]. A meta-analysis of approximately 1000 patients identified clonal TMB, CXCL9 expression and other factors as independent predictors of ICI response [277], and the “cancer immunogram” provided a conceptual framework for combining tumor foreignness, immune status and host factors [278]. Clinical models built on routinely available variables, such as TMB, albumin, NLR, age and cancer type, outperformed TMB alone in retrospective and external validation cohorts [279,280]. Their reliance on cheap, widely available data makes them attractive, but they have not yet been validated prospectively (Tier 4).

Artificial intelligence (AI) and machine learning are increasingly used to integrate multi-omics, imaging and clinical data to predict response, resistance and toxicity [27,281,282]. Only a handful of AI models, however, have external or blinded independent validation, with MSI prediction from hematoxylin and eosin (H&E) slides being the clearest example [283,284]. Most models that predict ICI response have been validated only retrospectively [285] and should be treated as exploratory until prospective data are available (Section 11.4).

### 8.4. Microbiome Profiling Technologies

Microbiome studies use either 16S rRNA gene sequencing, which profiles taxa at the genus level, or shotgun metagenomics, which resolves species and strains and also captures functional genes. Early cohorts linked taxa such as *Akkermansia muciniphila*, *Bifidobacterium* and *Faecalibacterium* spp. to response to anti-PD-1 therapy [286,287,288], and fecal microbiota transplantation from responders restored responses in some patients with anti-PD-1-refractory melanoma [289,290]. A cross-cohort analysis, however, found that microbial signatures of response do not replicate consistently across populations [291]. Standardized stool collection, sequencing and analysis are therefore needed, as is validation of function-based rather than taxon-based signatures. At present, recent antibiotic exposure, which is associated with worse outcomes on ICIs [214], is the most clinically actionable microbiome-related factor.

### 8.5. Extracellular Vesicle-Based Technologies

EVs are isolated by ultracentrifugation, size-exclusion chromatography, precipitation, immunoaffinity capture or microfluidic devices and characterized by nanoparticle tracking, flow cytometry and proteomic or RNA profiling; the MISEV2023 recommendations set out how such work should be reported [292]. Clinically, circulating exosomal PD-L1 suppresses T-cell function, and an early rise during pembrolizumab therapy distinguished responders from non-responders in melanoma [117]; tumor-derived exosomal PD-L1 also promotes systemic immunosuppression [118]. EV-based assays nonetheless remain exploratory (Tier 4), because different isolation methods give different yields and purities and large validation studies have not yet been done.

## 9. Biomarker-Guided Immunotherapy Across the Treatment Course

### 9.1. Patient Selection

Before treatment starts, biomarkers decide eligibility and regimen: PD-L1 TPS or CPS for PD-(L)1 inhibitors alone or in combination, MSI-H/dMMR and TMB-H for tissue-agnostic use, EGFR/ALK status to favor targeted therapy over ICIs in NSCLC, and HLA type plus target antigen expression for TCR-based therapies (Table 1). In dMMR rectal cancer, selection by biomarker has even made organ-sparing treatment with PD-1 blockade alone possible [67].

### 9.2. Treatment Response Assessment

Imaging is an imperfect guide to ICI response because responses can be delayed and pseudoprogression occurs. Early ctDNA kinetics offer a complementary readout: clearance or a sharp fall in ctDNA during the first weeks of treatment is associated with radiological response and longer survival and can help separate pseudoprogression from true progression [87,88,293]. Reinvigoration of peripheral T cells relative to tumor burden provides an early pharmacodynamic signal [85].

### 9.3. Resistance Monitoring

Repeated tissue or liquid biopsies can reveal acquired resistance, for example new B2M or JAK1/2 alterations after ICIs [136] or loss of the target antigen after CAR-T therapy [236], and may prompt a switch to a strategy that does not depend on the lost pathway.

### 9.4. Treatment Adaptation

Biomarkers can also support escalation or de-escalation. In an exploratory analysis of IMvigor010, patients with muscle-invasive urothelial carcinoma who were ctDNA-positive benefited from adjuvant atezolizumab, whereas ctDNA-negative patients did not [294], an observation that led to prospective ctDNA-guided adjuvant trials. In hematological malignancies, MRD status guides the use of blinatumomab [89], and serial cytokine measurements after CAR-T or bispecific therapy help time the use of tocilizumab and corticosteroids.

### 9.5. Technologies for Dynamic Biomarker Monitoring

Several technologies allow repeated, minimally invasive monitoring, including serial ctDNA analysis, high-dimensional cytometry of blood [266] and molecular imaging. In one study, uptake of ^89^Zr-labeled atezolizumab on positron emission tomography predicted response better than PD-L1 status by IHC [295]. Preclinical work has produced peptide-based biosensors for sensitive detection of tumor immunology-related targets [296] and engineered systems that allow spatiotemporal control and readout of immune checkpoint molecules on cells [297]. Supramolecular peptide nanoprobes can image immune checkpoint blockade (ICB)-enhanced ferroptosis as it happens [298], and multifunctional peptides that block checkpoints while reprogramming the immunosuppressive microenvironment or enhancing ferroptosis combine monitoring with therapy [41,299]. These theranostic tools are still in the preclinical stage (Tier 4) and point toward real-time tracking of checkpoint engagement and immunotherapy-induced tumor cell death, which could eventually allow treatment to be adjusted earlier.

## 10. Challenges and Limitations of Biomarkers in Cancer Immunotherapy

Immunotherapy biomarkers have come a long way, but obstacles still limit their practical application [300]. Analysing the challenges will help enhance the reliability, reproducibility, and overall utility of biomarkers [301]. Tumors exhibit diversity at the genetic, transcriptional, and immunological levels, showing both intra-tumoral heterogeneity (within a single tumor) and inter-tumoral heterogeneity (across multiple tumors within a single patient) [302]. This variability may result in inconsistent expression of biomarkers and adversely influence the predictive value of single-site biopsies [303]. PD-L1 expression can vary markedly across multiple areas of a single tumor, leading to inaccurate categorization of patients who qualify for immune checkpoint inhibitor therapy [304]. Not having standardized formats, scoring methods, biomarker assays, and cutoff values reduces reproducibility and makes cross-study comparison of results difficult [305]. There is substantial heterogeneity across platforms, reagents, and criteria for interpreting PD-L1 testing, TMB assessment, and immune gene expression profiling [108]. The enhancement of clinical utility has necessitated standardizing approaches and creating consensus-based guidelines [306]. Changes in biomarker levels could result from changes in the effects of a treatment, the progression of a disease, and/or changes in immune system modulation [102]. For example, changes in tumor–immune interactions, ctDNA, cytokine profiles, and immune cells in the tumor microenvironment can occur due to therapy [307]. The use of single-point measurements may omit critical aspects of immune response evolution, indicating a need for longitudinal tracking [308]. More sophisticated biomarker evaluation, such as multi-omics profiling, single-cell sequencing, and liquid biopsies, tends to be costly and consume an exorbitant amount of resources [309]. The lack of high-throughput technologies in most clinical settings may also limit fair access to therapies guided by biomarkers [310]. Moreover, the intricate analysis of such data requires additional cost and logistical barriers in terms of specialized bioinformatics expertise [311]. All biomarkers have trade-offs in terms of sensitivity and specificity. False-positive outcomes can preemptively expose patients to unnecessary, potentially toxic treatments, while false negatives can deny patients access to beneficial treatments [312]. An example of the limitations of single biomarker reasoning is that patients with low PD-L1 expression can still respond to PD-1/PD-L1 inhibitors. To avoid such pitfalls, the use of multiple biomarkers, or composite scoring, should be considered [313].

## 11. Future Perspectives

Cancer immunotherapy continues to focus on developing robust, clinically actionable biomarkers to improve personalized treatment [102]. Newer [102] approaches aim to improve biomarker predictive precision, enable real-time monitoring, and bring biomarkers to the forefront of everyday clinical decision-making [314].

### 11.1. Composite Biomarkers

Single biomarkers tend to miss many of the complexities of tumor–immune interactions [315]. The ability [316] to predict outcomes is enhanced by composite biomarkers, which integrate various molecular, cellular, and systemic components [316]. A composite [315] biomarker consists of multiple biological, cellular, and systemic components. They enhance the predictive capacity of the said biomarkers [25]. Integrative expressions involving tumor intrinsic biomarkers such as PD-L1 and TMB, tumor microenvironment biomarkers, such as TILs, immune signatures, and blood-based biomarkers, including ctDNA and cytokines, provide an opportunity for enhanced patient stratification and targeting therapy selection [316]. The main biomarker categories are summarized in Table 2 [316].

### 11.2. Personalized Immunotherapy

Recent developments in genomics, proteomics, and neoantigen discovery are paving the way for advancements in personalized immunotherapy [317]. Tailored therapies targeting specific tumor mutation profiles, immune microenvironments, and microbiome details enhance effectiveness while minimizing adverse effects [210]. For example, vaccines targeting neoantigens and adoptive T-cell therapies can be personalized to a specific patient’s tumor mutations, thereby improving immune recognition and clinical benefits [318].

### 11.3. Dynamic Monitoring and Clinical Integration

The monitoring of treatment response, treatment resistance, and immune-related adverse event responses can be assessed using longitudinal dynamic biomarker approaches [21]. Methods associated with liquid biopsies, such as circulating immune cell analysis, cell-free protein biomarker assessments, and ctDNA analysis, provide a minimally invasive way to monitor the immune response and tumor burden during treatment [319]. Continuous monitoring aids adaptive modification of treatment plans, anticipatory steps to prevent adverse events, and rapid detection of resistance mechanisms [320]. For biomarkers to affect patient care, they must be integrated into the clinical decision-making process, meet standards and undergo validation, and be incorporated into clinical workflow [321]. The addition of high-throughput technologies, computational modeling, and artificial intelligence-based predictive technologies will assist in selecting personalized therapies, stratifying risk, and predicting outcomes [322]. To create guidelines grounded in evidence and to prioritize biomarker-directed immunotherapy for a wide range of patients, collaboration among clinicians, researchers, and regulatory bodies is needed [310].

### 11.4. Artificial Intelligence in Immunotherapy Biomarker Development

AI is likely to affect biomarker development in several ways. Computational pathology can extract from routine H&E slides information that currently requires separate tests: deep-learning models predict MSI status directly from H&E images [323], have been validated externally in multicenter cohorts [283], and one such tool (MSIntuit) has been validated in a blinded independent cohort as a pre-screening test that could reduce confirmatory molecular testing [284]. Similar approaches are being developed to automate PD-L1 TPS/CPS scoring and TIL counting and so reduce inter-observer variability. Radiomics can estimate the immune phenotype non-invasively; a CT-based signature of CD8^+^ T-cell infiltration, for instance, was associated with response to anti-PD-1/PD-L1 therapy in a retrospective multicohort study [324]. Multimodal models that combine radiology, pathology and genomics [325], or routine clinical, laboratory and genomic variables [279,280], have outperformed PD-L1 or TMB alone in retrospective and external validation cohorts, and transcriptome-based models such as TIDE, which capture T-cell dysfunction and exclusion, follow the same logic [326]. Longitudinal models that combine serial ctDNA, peripheral immune profiles and electronic health records could support dynamic prediction of response, resistance and immune-related adverse events, and foundation models trained on large collections of pathology images and omics data may make it possible to build biomarker models from small, well-annotated immunotherapy cohorts.

For now, the evidence is mostly retrospective, and to our knowledge no AI-derived predictor of immunotherapy benefit has been approved as a companion diagnostic [285]. Before such models reach the clinic, they should be developed on data with standardized pre-analytical handling and scanning or imaging protocols; locked before testing in fully independent external cohorts; evaluated prospectively, ideally in biomarker-stratified or randomized designs; reported according to TRIPOD+AI and assessed in clinical studies according to DECIDE-AI and CONSORT-AI/SPIRIT-AI [327,328,329]; checked for bias across sex, ancestry and care settings, with outputs that clinicians can interpret; and brought into line with regulatory requirements for software as a medical device, including post-market performance monitoring. Federated learning and data-sharing consortia could help assemble the large and diverse datasets needed while protecting patient privacy.

**Table 2:** Overview of Biomarkers in Cancer Immunotherapy.

Category	Key Components	Clinical Relevance	References
Cancer Immunotherapy Overview	Immune checkpoint inhibitors, CAR-T cells, and cancer vaccines.	Harnesses the immune system to eliminate tumor cells and improve survival outcomes.	[1,6]
Challenges in Immunotherapy	Non-response, acquired resistance, and tumor heterogeneity.	Limits therapeutic efficacy; necessitates biomarker-guided approaches.	[330,331]
Tumor-Intrinsic Biomarkers	PD-L1 expression, tumor mutational burden (TMB), microsatellite instability (MSI), and neoantigens.	Predict response to immunotherapy; guide patient selection.	[332,333]
Tumor Microenvironment Biomarkers	Tumor-infiltrating lymphocytes (TILs), cytokines, and immune cell signatures.	Reflect immune activation and tumor–immune interactions.	[334,335]
Circulating Biomarkers	Circulating tumor DNA (ctDNA), circulating immune cells, and exosomes.	Enable non-invasive, real-time monitoring of treatment response.	[336,337]
Systemic Biomarkers	Microbiome composition, metabolic profiles.	Influence immune modulation and therapeutic outcomes.	[338,339]
Emerging Technologies	Multi-omics, single-cell sequencing, and artificial intelligence.	Enhance biomarker discovery and precision medicine approaches.	[340,341]
Dynamic Monitoring	Longitudinal biomarker assessment (e.g., ctDNA, cytokines).	Tracks treatment response; detects resistance early.	[342,343]
Clinical Applications	Patient stratification, response prediction, and toxicity monitoring.	Supports personalized immunotherapy strategies.	[50,344]
Future Directions	Composite biomarkers, real-time integration, and resistance mechanism identification.	Improves the accuracy and clinical utility of biomarkers.	[345,346]

## 12. Conclusions

Judged against analytical validity, clinical validity, clinical utility and regulatory readiness, only a handful of immunotherapy biomarkers are ready to guide treatment today: PD-L1 IHC with assay- and indication-specific cut-offs, MSI-H/dMMR status, TMB-H measured with an approved NGS assay, HLA type and target antigen expression for TCR-based therapies, and, in hematological malignancies, target antigen expression and MRD. Each is tied to a particular therapy, cancer type, assay and cut-off, and none cleanly separates responders from non-responders.

A second group of markers has a strong biological rationale and clinical validation but no proven clinical utility. It includes tumor-informed ctDNA for MRD detection and early response assessment, TLS, T-cell-inflamed gene expression profiles, spatial immune phenotypes, CAR-T product and expansion metrics, and pre-infusion toxicity scores. Resistance-associated alterations (B2M, HLA LOH, JAK1/2, STK11/KEAP1), microbiome and metabolite signatures, extracellular vesicles, soluble cytokines and most AI-derived models remain exploratory, and non-specific inflammatory markers such as IL-6, CRP and ferritin are useful for monitoring toxicity but should not be read as validated predictors.

In our view, the main obstacles to clinical use are methodological rather than biological. Candidate markers usually fail because pre-analytical handling is not standardized, assays are not harmonized, cut-offs are chosen after the data are seen, independent validation is missing, or there is no prospective evidence that acting on the marker improves outcomes. Box 1 summarizes the steps needed to close these gaps. The most promising direction is probably composite, dynamic strategies that pair a validated tissue marker with serial blood-based monitoring and are tested prospectively in biomarker-stratified trials.

Box 1 **Roadmap from candidate biomarker to clinical use.**

1. Clinical question and intended use

Define the therapy, cancer type, setting and decision the biomarker will inform (selection, exclusion, monitoring, toxicity).

2. Standardized pre-analytical processing

Specify sample type, collection timing, cold ischemia and fixation time, tubes and plasma processing, storage and minimum tumor content.

3. Analytical validation

Establish accuracy, precision, limit of detection, linearity and reproducibility across operators, instruments and laboratories (ring trials); harmonize assays (e.g., the Blueprint PD-L1 project and the TMB Harmonization Project) [121,347].

4. Pre-defined clinical cut-off

Lock the cut-off and, for models, the algorithm, before validation; avoid choosing cut-offs after seeing the data.

5. Independent validation

Confirm clinical validity in independent, external cohorts, reported according to REMARK (biomarkers) or TRIPOD+AI (prediction models) [327,348]; distinguish predictive from prognostic effects using randomized data.

6. Prospective validation

Use biomarker-stratified, enrichment or biomarker-strategy trials, or prospective–retrospective analyses of archived trial specimens [48].

7. Proof of clinical utility

Show that biomarker-guided management improves survival, reduces toxicity or avoids ineffective treatment, with health-economic evaluation.

8. Regulatory approval and implementation

Obtain companion or complementary diagnostic approval or guideline endorsement; ensure access, quality assurance and post-market real-world monitoring.

## Figures and Tables

**Table 1:** Evidence hierarchy of biomarkers for cancer immunotherapy.

Biomarker	Cancer Type	Treatment	Assay/Sample Type	Cut-Off	Intended Use	Level of Validation	Major Limitations
**Tier 1—Regulatory-approved/companion diagnostic**							
PD-L1 TPS	NSCLC	Pembrolizumab (and other PD-(L)1 inhibitors)	IHC (22C3; also 28-8, SP263); FFPE tumor tissue	TPS ≥50% (first-line monotherapy); ≥1%	Patient selection	Tier 1 (CDx)	Spatial and temporal heterogeneity; differences between assays and platforms; responses also occur in PD-L1-negative tumors
PD-L1 CPS	HNSCC, gastric/GEJ, esophageal, cervical, TNBC	Pembrolizumab, nivolumab	IHC (22C3, 28-8); FFPE	CPS ≥1, ≥5 or ≥10 depending on indication	Patient selection	Tier 1 (CDx)	Cut-offs differ by indication and assay; inter-observer variability in immune-cell scoring
PD-L1 TC/IC	NSCLC, urothelial carcinoma	Atezolizumab, durvalumab	IHC (SP142, SP263); FFPE	Assay-specific tumor-cell/immune-cell thresholds	Patient selection	Tier 1 (CDx)	SP142 stains fewer tumor cells than other assays; results are not interchangeable
MSI-H/dMMR	Tissue-agnostic; colorectal, endometrial, others	Pembrolizumab, dostarlimab, nivolumab ± ipilimumab	MMR IHC (MLH1, MSH2, MSH6, PMS2), PCR or NGS; FFPE	Loss of ≥1 MMR protein/MSI-H	Patient selection; Lynch syndrome screening	Tier 1 and Tier 2 (universal testing in colorectal and endometrial cancer)	IHC/PCR discordance in non-colorectal tumors; primary resistance in a subset of patients
TMB-H	Solid tumors (tissue-agnostic)	Pembrolizumab	NGS panel (FoundationOne CDx); FFPE	≥10 mut/Mb	Patient selection after prior therapy	Tier 1 (accelerated approval)	Variation between panels and bioinformatic pipelines; a single cut-off performs poorly in some histologies
HLA-A*02:01 genotype	Metastatic uveal melanoma	Tebentafusp	HLA typing; blood	HLA-A*02:01-positive	Eligibility	Tier 1	Excludes HLA-ineligible patients; no predictor of benefit among eligible patients
HLA-A*02 + MAGE-A4	Synovial sarcoma	Afamitresgene autoleucel	HLA typing (blood) + MAGE-A4 IHC (tissue)	Eligible HLA-A*02 alleles; MAGE-A4 ≥2+ in ≥30% of tumor cells	Eligibility	Tier 1	Small, restricted population; antigen heterogeneity
**Tier 2—Guideline-recommended/used in routine practice**							
CD19/BCMA expression	B-cell malignancies; multiple myeloma	CAR-T, bispecific engagers	Flow cytometry/IHC; blood, marrow, tissue	Presence (no validated density threshold)	Target confirmation, especially at relapse	Tier 2	Antigen loss or low antigen density at relapse; density not standardized
IL-6, CRP, ferritin	Hematological malignancies	CAR-T, bispecific engagers	Serum	No validated thresholds	Monitoring of CRS/HLH-like toxicity	Tier 2 (monitoring only)	Non-specific (infection, inflammation); not validated as pretreatment predictors
LDH	Melanoma	All systemic therapies	Serum	> upper limit of normal	Prognosis/staging (AJCC M category)	Tier 2	Prognostic, not predictive; non-specific
MRD	B-ALL	Blinatumomab	Flow cytometry/PCR/NGS; bone marrow	≥10^−3^ (approved indication)	Patient selection; response monitoring	Tier 1–2	Limited to hematological malignancies
**Tier 3—Clinically validated but not routinely used**							
Tumor-informed ctDNA	Urothelial, NSCLC, melanoma, pan-cancer	ICIs	Plasma NGS/ddPCR	Detectable vs. undetectable; early-on treatment decline	MRD; early response; adjuvant treatment decisions	Tier 3 (prospective ctDNA-guided trials ongoing)	Low-shedding tumors; pseudoprogression; cost; no standardized kinetic thresholds
IFN-γ/T-cell-inflamed GEP	Pan-tumor	Anti-PD-1	RNA expression panel; FFPE	Trial-specific score	Response prediction	Tier 3	No approved assay or cut-off; overlaps with PD-L1
Stromal TILs; Immunoscore	Breast cancer (TNBC); colon cancer	Prognosis (not ICI selection)	H&E; CD3/CD8 IHC with digital analysis	Standardized scoring methods	Prognosis	Tier 3 (prognostic)	Not validated for selecting ICI treatment
Tertiary lymphoid structures	Soft-tissue sarcoma, melanoma, RCC	Anti-PD-1	H&E/IHC; RNA signatures	Presence of mature TLS	Patient selection	Tier 3 (prospective enrichment cohort)	No standard definition of maturity or scoring method
Blood TMB	NSCLC	Atezolizumab	Plasma NGS	≥16 mut/Mb (trial-specific)	Patient selection	Tier 3 (prospective validation cohort did not meet its primary endpoint)	Depends on ctDNA fraction; no standard cut-off
CAR-HEMATOTOX; modified EASIX	LBCL, myeloma	CAR-T	Routine laboratory values	Score-specific	Toxicity risk stratification	Tier 3 (retrospective external validation)	Retrospective development; not linked to proven preventive interventions
AI-based MSI pre-screening from H&E	Colorectal cancer	Selection for ICIs (via MSI)	Whole-slide H&E images	Model-specific	Pre-screening before molecular confirmation	Tier 3 (blinded independent validation)	Requires confirmatory testing; sensitive to scanner and staining differences
**Tier 4—Exploratory/preclinical**							
B2M, JAK1/2, HLA LOH, STK11/KEAP1, WNT/β-catenin	Melanoma, NSCLC, others	ICIs	NGS; FFPE tissue	Presence of alteration	Prediction of resistance	Tier 4 (retrospective)	Predictive vs. prognostic effect unclear; confounding by co-mutations
Spatial metrics (mIHC/IF, spatial transcriptomics); TCR clonality	Multiple	ICIs	FFPE multiplex imaging; TCR sequencing (tissue or blood)	None established	Response prediction	Tier 4	Platform-specific; non-harmonized analysis pipelines
Gut microbiome composition	Melanoma, NSCLC, RCC	ICIs	Stool 16S rRNA/shotgun metagenomics	None	Prediction; therapeutic modulation (FMT)	Tier 4	Signatures inconsistent across cohorts; diet, geography and antibiotic exposure confound results
Metabolites (kynurenine, lactate, adenosine)	Multiple	ICIs	Plasma/tumor mass spectrometry	None	Prediction	Tier 4	Pre-analytical sensitivity; affected by diet and drugs
Exosomal PD-L1	Melanoma, NSCLC	Anti-PD-1	Plasma extracellular vesicles	None	Prediction; early response	Tier 4	Isolation and characterization methods not standardized
Soluble PD-L1; serum cytokines; NLR	Multiple	ICIs	Serum/complete blood count	Variable	Prognosis	Tier 4	Non-specific; inconsistent cut-offs across studies
Multimodal/clinical-genomic AI models	Pan-cancer, NSCLC	ICIs	Clinical, laboratory, genomic, imaging and pathology data	Model-specific	Response prediction	Tier 4 (retrospective external validation only)	No prospective validation; risk of bias; differences across sites

AI, artificial intelligence; B-ALL, B-cell acute lymphoblastic leukemia; BCMA, B-cell maturation antigen; CDx, companion diagnostic; CPS, combined positive score; CRP, C-reactive protein; CRS, cytokine release syndrome; ctDNA, circulating tumor DNA; ddPCR, droplet digital PCR; FFPE, formalin-fixed paraffin-embedded; FMT, fecal microbiota transplantation; GEJ, gastroesophageal junction; GEP, gene expression profile; H&E, hematoxylin and eosin; HLA, human leukocyte antigen; HLH, hemophagocytic lymphohistiocytosis; HNSCC, head and neck squamous cell carcinoma; IC, immune cells; ICI, immune checkpoint inhibitor; IHC, immunohistochemistry; IL-6, interleukin-6; LBCL, large B-cell lymphoma; LDH, lactate dehydrogenase; LOH, loss of heterozygosity; mIHC/IF, multiplex immunohistochemistry/immunofluorescence; MMR, mismatch repair; MRD, measurable residual disease; NGS, next-generation sequencing; NLR, neutrophil-to-lymphocyte ratio; RCC, renal cell carcinoma; TC, tumor cells; TLS, tertiary lymphoid structures; TMB, tumor mutational burden; TNBC, triple-negative breast cancer; TPS, tumor proportion score.

## Data Availability

No new data were created or analyzed in this study. Data sharing is not applicable to this article.

## Abbreviations

The following abbreviations are used in this manuscript:

AI	Artificial intelligence
ASTCT	American Society for Transplantation and Cellular Therapy
AUC	Area under the curve
B2M	β2-microglobulin
BCMA	B-cell maturation antigen
CAR-NK	Chimeric antigen receptor natural killer cell
CAR-T	Chimeric antigen receptor T cell
CPS	Combined positive score
CRP	C-reactive protein
CRS	Cytokine release syndrome
ctDNA	Circulating tumor DNA
CTLA-4	Cytotoxic T-lymphocyte-associated antigen-4
dMMR	Mismatch repair deficiency
ER	Endoplasmic reticulum
EV	Extracellular vesicle
FDA	U.S. Food and Drug Administration
FMT	Fecal microbiota transplantation
H&E	Hematoxylin and eosin
HIF-1α	Hypoxia-inducible factor-1α
HLA	Human leukocyte antigen
ICANS	Immune effector cell-associated neurotoxicity syndrome
ICI	Immune checkpoint inhibitor
IFN-γ	Interferon-γ
IHC	Immunohistochemistry
IL	Interleukin
irAEs	Immune-related adverse events
LDH	Lactate dehydrogenase
LOH	Loss of heterozygosity
MDSCs	Myeloid-derived suppressor cells
MHC	Major histocompatibility complex
mIHC/IF	Multiplex immunohistochemistry/immunofluorescence
MRD	Measurable (minimal) residual disease
MSI	Microsatellite instability
MSI-H	Microsatellite instability-high
NGS	Next-generation sequencing
NK	Natural killer
NLR	Neutrophil-to-lymphocyte ratio
NSCLC	Non-small cell lung cancer
PD-1	Programmed cell death protein-1
PD-L1	Programmed death-ligand 1
scRNA-seq	Single-cell RNA sequencing
TAMs	Tumor-associated macrophages
TCR	T-cell receptor
TGF-β	Transforming growth factor-β
TILs	Tumor-infiltrating lymphocytes
TLS	Tertiary lymphoid structures
TMB	Tumor mutational burden
TME	Tumor microenvironment
TNF	Tumor necrosis factor
TNF-α	Tumor necrosis factor-α
TPS	Tumor proportion score
Tregs	Regulatory T cells
